# A Critical Review on the Junction Temperature Measurement of Light Emitting Diodes

**DOI:** 10.3390/mi13101615

**Published:** 2022-09-27

**Authors:** Ceren Cengiz, Mohammad Azarifar, Mehmet Arik

**Affiliations:** 1 Department of Mechanical Engineering, Virginia Polytechnic Institute and State University, Blacksburg, VA 24061, USA; 2 Department of Mechanical Engineering, Auburn University, Auburn, AL 36849, USA; 3 Evateg Center, Ozyegin University, Istanbul 34794, Turkey

**Keywords:** junction temperature, light emitting diode (LED), heat generation, review, measurement systems, thermometry

## Abstract

In the new age of illumination, light emitting diodes (LEDs) have been proven to be the most efficient alternative to conventional light sources. Yet, in comparison to other lighting systems, LEDs operate at low temperatures while junction temperature (*T_j_*) is is among the main factors dictating their lifespan, reliability, and performance. This indicates that accurate measurement of LED temperature is of great importance to better understand the thermal effects over a system and improve performance. Over the years, various *T_j_* measurement techniques have been developed, and existing methods have been improved in many ways with technological and scientific advancements. Correspondingly, in order to address the governing phenomena, benefits, drawbacks, possibilities, and applications, a wide range of measurement techniques and systems are covered. This paper comprises a large number of published studies on junction temperature measurement approaches for LEDs, and a summary of the experimental parameters employed in the literature are given as a reference. In addition, some of the corrections noted in non-ideal thermal calibration processes are discussed and presented. Finally, a comparison between methods will provide the readers a better insight into the topic and direction for future research.

## 1. Introduction

The temperature rise has been an indispensable part of light generation systems. This might explain why we continue to distinguish visible light colors by the temperature of an ideal black-body radiator of a color comparable to that of the light source [1]. Thermal challenges of light generation systems date back to 125,000 years ago as anatomically modern human beings controlled fire for sources of heating and illumination [2,3], dealing with torches, cressets, and bonfires which burnt at near 1000 °C [4]. Illumination relied on the control of flame and burning fuels created from olive and seed oils, animal grease, tallow, and gas till the 18th century [5]. In the 19th century, rapid progress in electrical science laid the foundation of modern progress in lighting [6,7]. Still, temperature rise was a major challenging part of light generation. From 1835 after the first constant electric light demonstration by Charles Wheatstone, it took 40 years for scientists to figure out how to prevent the 3000 K filament from oxidizing [8]. Interestingly, from 1924 to 1940, as the lifetime of lightbulbs started to exceed 1800 h, financial agreements led to thermally controlled life-span engineering for added profits [9]. In 1986, the invention of the first nitride-based blue light emitting diode (LED) by Isamu Akasaki and others eventually revolutionized the lighting industry, allowing high power and efficient solid state lighting [10]. This breakthrough followed by a rapid optimization process in LED device production led to a cost/lumen reduction of 10-fold and a lumen per LED package increment of 20-fold in each decade [11]. In terms of efficacy, fluorescent lamps, despite being able to operate at almost room temperature and possibly having the longest lifespan, cannot compete with LEDs [12]. In addition, LEDs have shown the potential to go beyond the “replacement paradigm” by providing a critical advantage in transportation, display and imaging systems, communications, agriculture, and other fields with their advanced color control and modulation capabilities [13,14].

The wavelength of the emission could be engineered with band gap control of the materials in the diffusion region between the p-type and n-type regions of an LED, within a few microns near the p-n junction (λ = 1240/E_g_ nm) [15]. LEDs are now capable of photon emission in all visible light wavelengths by progressing from ternary (GaAs_1-x_P_x_) material selection to quaternary compositions (AlGaInN). AlGaAs material systems are used for infrared and red emission, AlGaInP for amber, orange, and yellow-green, and AlInGaN for green to near ultraviolet. Because of their high thermal conductivity, electron saturation drift velocity, critical breakdown voltage, and fracture toughness to resist defects growth, group III nitride materials are ideal for unlocking the higher powers in LEDs [16,17]. However, given the fraction of input power of previous lighting technologies and the highest power conversion efficiency of any known man-made light source, why is it challenging to keep them working at low temperatures?

**Figure 1:**
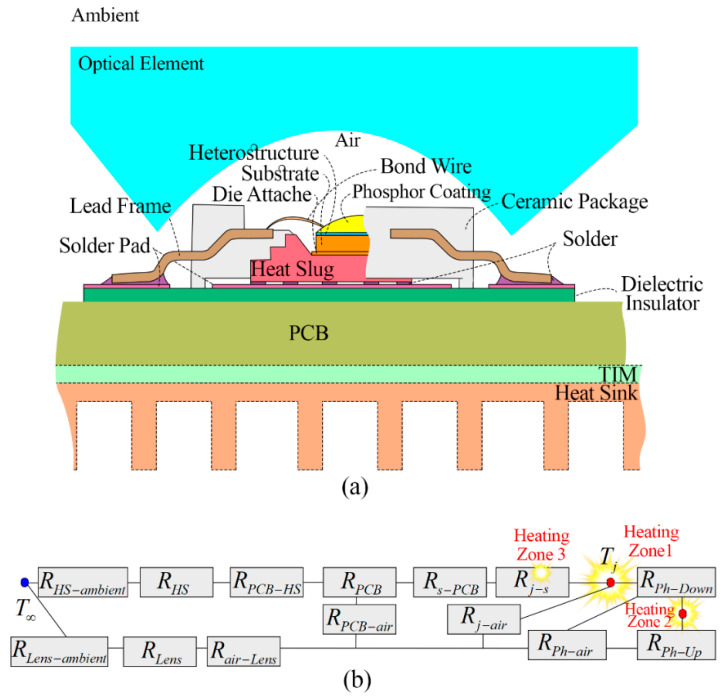
(**a**) Schematic overview of the package and structure of a typical LED luminaire and corresponding (**b**) thermal resistance network with main heat generation zones indicated by heat generating zones 1 to 3. R_j-s_ and R_j-air_ indicate thermal resistance from the junction to the solder point and to air (trapped air inside the luminaire), respectively. R_s-PCB_ indicates thermal resistance introduced from package mount to PCB. R_PCB_, R_PCB-HS_, and R_PCB-air_ are thermal resistance of the package, interfacial thermal resistance from PCB to heat sink, and convection resistance from PCB to air (air trapped in the luminaire), respectively. R_HS_ and R_HS-ambient_ are the thermal resistance of the attached heat sink and the convection from the heat sink to the ambient air, respectively. In case of having phosphor coating, R_Ph-Down_ and R_Ph-up_ are thermal resistances in the phosphor coating (phosphor generates heat due to thermal quenching and Stokes shift). R_air-Lens_, R_Lens_, and R_Lens-ambient_ denote the thermal resistance of hot trapped air to the covering lens, the thermal resistance of the lens itself, and the convection from lens to ambient, respectively.

LEDs (along with all other power semiconductor devices) act as discrete heat sources, dissipating heat through electronic packages into printed circuit boards (PCBs) and heat sinks. Currently, in optimum current densities of LEDs, around 70% of the input electrical power converts into heat generation [18]. Figure 1a schematically shows the structure of a sample lead frame with a metal/plastic body package [19]. As shown in Figure 1b, the primary cause of the temperature rise in LEDs is the stacked thermal resistance barriers in their complex thermal architecture [20,21]. Junction temperature (*T_j_*) is the largest recommended operating temperature in an electronic structure, which is shown in the thermal resistance network in Figure 1b. As indicated by the heat generating zones (1 to 3) in the thermal resistance map of a typical LED in Figure 1b, heat is being generated in an LED at (1) the active region due to non-radiative recombination, inside of the optical cavity due to radiation absorption, electrical structures and semiconductor metal interfaces due to Joule heating [22], (2) phosphor–polymer composite/coating in a white LED due to Stokes shift and thermal quenching of phosphor [23,24,25,26,27], and (3) outside of the optical cavity due to the radiation absorption and other electrical structures due Joule heating [28,29]. Currently, chip on board, wafer level, and chip-scale packaging are the most popular designs in the LED market for heat dissipation [30]. In the past three decades, the thermal resistance of the packages has reduced from 8 K/W [31] in 2008 to 5 K/W [32] in 2016 and now to 3.5 K/W [33].

Besides the compact size, the challenge of reducing the thermal resistance of an LED package is mainly related to the scarcity of suitable substrate materials where chips are grown heteroepitaxially on foreign sapphire and SiC substrates with a large lattice and thermal expansion coefficient mismatch (future candidates may be hexagonal boron nitride substrate with plasma enhanced chemical vapor deposition (PECVD) and amorphous boron nitride substrate prepared by magnetron sputtering have been reported in AlGaN/GaN high electron mobility transistors [34,35,36]). Additional interfacial thermal resistance between each layer also increases the total thermal resistance of the package [21,37,38]. These types of integrations, as shown in Figure 1b, limit the heat dissipation capability of the package due to the introduction of interfacial thermal resistances where the thermal characterization techniques are rarely investigated [39]. Attachment of a package to a PCB can introduce additional three-dimensional thermal resistance (up to 50 K/W [40,41]) where the solder joint quality and thermomechanical properties of the package can also restrain the heat dissipation. Furthermore, there is another heat dissipation barrier from the PCB to any additional heatsink and to the external environment (up to 30 W/K depending on geometry and convective characteristics [40,41]) and also from the phosphor layer to the lens [29,42].

The temperature rise of the chip influences the luminance performance of the LED and temperature-induced failure modes [43]. The aging and degradation of LEDs, the majority of which is caused by high temperatures, has long been a topic of debate [44,45,46,47]. It is recognized that the temperature of the junction region and phosphor layer (in phosphor-converted white LEDs) are the two major thermal concerns for next generation, high power LED devices [48,49,50], and successful thermal management was found to be the key aspect to tackle thermal-induced problems [51,52]. However, to offer a suitable thermal management solution, interpreting photometric characteristics and package capabilities and making lifetime predictions, it is required to adopt a steady-state and transient temperature characterization in a large range of conditions.

Over the last 20 years, a number of *T_j_* measurement techniques have been introduced by researchers, and they have progressed in many ways. Yet, it remains as a hot topic since researchers are frequently coming up with new approaches to determine the *T_j_* or making improvements upon the existing techniques. Currently, measurements based on temperature-dependent variation of optical or electrical properties or physical contact are commonly employed techniques. However, it is important to note that experimental *T_j_* measurement of LEDs is not a straightforward task. The majority of the LED modules comprise surface covering lens, encapsulation, and other package components that obstruct the chip temperature measurements. This highlights the fact that the selection of appropriate techniques may be limited by the unique requirements and operating conditions of the inspected device.

There have been some reviews that presented general temperature measurement techniques [53], temperature measurement of semiconductors [54,55], and various electronic modules [56,57,58,59]. However, to the best of our knowledge, an extensive review that focuses explicitly on experimental *T_j_* measurement of LEDs has yet to be completed. Correspondingly, we aimed to put an emphasis on the various experimental measurement techniques that are available to assess the junction temperature of LEDs. This review is composed of a large number of investigations on *T_j_* of LEDs and gives detailed discussions of the advantages and disadvantages of each technique based on findings presented in the literature. The selection of experimental parameter reports of different researchers is given as a reference for comparison. The review starts by looking at temperature sensitive optical parameters (TSOPs) that are used for *T_j_* determination. In addition to the conventional approaches such as wavelength shift and broadening, new approaches are also covered. After which, optical temperature probing methods such as infrared thermography, thermoreflectance, Raman spectroscopy, and liquid crystal thermography are analyzed. Spatial and temporal resolutions, temperature sensitivity, and accuracy are taken into account when comparing these approaches. Then, temperature sensitive electrical parameters are investigated, and the primary focus has been given to the well-known forward voltage measurement (FVM) technique, while examples of other existing electrical temperature measurement methods used for LEDs are also mentioned. Finally, concluding remarks and a table summarizing the pros, cons, and future possibilities of various techniques are provided.

## 2. Temperature Sensitive Optical Parameters (TSOPs)

*T_j_* may be indirectly measured using an LED’s inherent optical characteristics. The emission spectrum of a semiconductor device is influenced by temperature variations due to the temperature dependence of the energy band gap [60]. This behavior motivates researchers to use spectral power distribution (SPD) characteristics such as the peak wavelength and spectral bandwidth of LEDs, which are known as temperature sensitive optical parameters (TSOPs), to estimate *T_j_* [61]. The TSOP measurement method is non-destructive and does not interrupt the electrical performance of LEDs [62,63,64,65,66] (e.g., in case of an alternating current (AC) LED [67], TSOP-based *T_j_* measurements have shown successful implementation without altering their electrical performance [68]).

**Figure 2:**
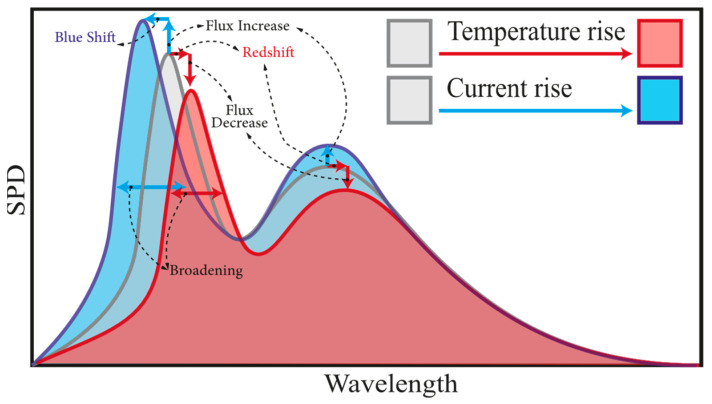
An illustration of temperature and input current induced changes in spectral power distribution (SPD) for an arbitrary WLED. At high temperatures, shown by the gray to red color transition in the figure, radiant flux reduction due to thermal droop, a red shift in dominant wavelengths of chip and phosphor, and full width at half maximum (FWHM) broadening are expected. At higher input current, shown in grey to blue color transition, an increase in power output, blue shift, and broadening at the chip’s FWHM can be seen.

The TSOPs are unique for each electrical working condition, and light output changes depending on the internal quantum efficiency and band gap characteristics of an LED at each specific temperature and input current [69]. A summarized illustration of the SPD response to temperature and input current induced changes of an arbitrary white LED (WLED) is shown in Figure 2.

It is known that with increasing *T_j_*, SPD shows a red shift and broadening [70]. The redshift phenomenon is primarily due to the band gap reduction. This was explained by Wang et al. [71,72] for GaN-based blue LEDs in low temperatures, and recently, similar results were reported at high temperatures for high brightness GaN on sapphire blue LEDs [73]. It should be noted that SPD displays a blue shift and broadening with increasing input current. Li et al. [74] studied the effect of input current and temperature on the spectral behavior of green InGaN/GaN multi-quantum well LED and showed that the excitation source could alter the carrier dynamics in the active region. A large blue shift was observed in high input power levels, mainly due to the carrier screening effect as a result of a weakened piezoelectric field that causes the quantum-confined Stark effect [75]. This issue can be verified by the findings of Kim et al. [76], which analyzed carrier leakage of GaN based on photoluminescence properties of LEDs both at forward biased and intentionally formed leakage path conditions. Increased current leakages were observed in low series resistance for LEDs, which led to a blue shift of the SPD.

Temperature-induced full width at half maximum (FWHM) broadening is due to the thermal broadening [77]. On the other hand, current-induced FWHM broadening is due to the combined effect of the screening to the piezoelectric field and band filling effect as discussed by Lin et al. [70]. With input currents of 150 to 850 mA for GaN-based blue LED at temperature range of 273 to 338 K, the slope of the center of mass wavelength per *T_j_* was below 0.034 nm/K, while for FWHM, broadening was above 0.052 nm/K. Assuming high precision temperature measurements by taking precise optical measurements within 0.1 nm, the accuracy of the *T_j_* measurement based on wavelength shift was around 3 K, while the FWHM measurement could reach below 2 K. Based on this, we suggest that FWHM calibration can yield high accuracy in *T_j_* evaluation.

It should be noted that the spectral width and shape of the LED emission can also be associated with carrier distribution, growth procedure and structure, the density of states, and the successful pairing of electrons and holes, etc. [78]. For instance, an inconsistent temperature-dependent shifting behavior in wavelength (red–blue–red shifting) was observed for the peak energy of an InGaN based MQW LED and mainly attributed to the non-uniformity (variation in layer thickness and defects) and carrier localization in quantum wells [79]. Considering these issues, the accuracy of the wavelength shift method is stated to be only 5–10% of the FWHM of the emission line [80]. However, in devices with a narrow emission, the accuracy of the wavelength shift method increases and can be a suitable approach for *T_j_* prediction [81].

Chhajed et al. [77] calibrated the peak wavelength of ultraviolet (UV), blue, green, and red GaN-based LEDs for a forward current range of 10 to 100 mA at temperatures of 22 to 120 °C. Similarly, a high temperature coefficient of the spectral widths was determined in comparison to the wavelength shift. Temperature coefficients of the dominant wavelength for the blue, green, and red LEDs were determined as 0.0389, 0.0308, and 0.1562 nm/K, respectively. The slopes increase to 0.0466, 0.0625, and 0.1812 nm/K for the FWHM-based *T_j_* evaluations. Similarly, a strong red shift of the red AlGaInP LED was also seen in another study by the authors for a trichromatic white LED system [82]. Blueshift was negligible for the red LED and slightly higher for the UV LED, while the highest blueshift effect was recorded for the green LED. The authors stated that the uncertainty of *T_j_* estimation based on the wavelength shift method is higher in comparison to the FWHM method. Furthermore, the authors preferred the forward voltage method (FVM) with a reported accuracy of ±3 °C over the TSOP method.

Chen et al. [83] conducted *T_j_* estimation experiments and obtained peak wavelength shifts for three different AlGaInP LED arrays. Shifting characteristics investigated at longer, central, and shorter wavelengths showed that the center wavelength is the most suitable method to calculate *T_j_* of an LED array. Tamura et al. [84] analyzed the wavelength shift of InGaN-based white LEDs at temperatures from 20 to 160 °C and their investigations showed that blue light emission from the active layer and yellow light emission from Ce:YAG phosphor formed two different electroluminescence bands while each band displayed a distinct behavior with the temperature change. However, similar to previous cases, *T_j_* was stated to be successfully calibrated to the blue emission of the chip. Chen et al. [85] showed a simplified peak wavelength shift variation at different *T_j_* for white LEDs under different drive currents. Their findings showed that the temperature dependence of peak wavelength is lower for direct current (DC) LEDs compared to bilevel drive, while the thermal energy needed for correlated color temperature (CCT) stabilization is also less for a DC LED. Gu et al. [86] selected the point of interest as the lowest energy in the SPD between the peaks of blue and yellow emissions. The ratio of the total radiant energy of white LEDs to the radiant energy within the blue emission in a different *T_j_* has shown a linear relationship. The authors claimed that with a ratio of 0.005, the temperature prediction accuracy of 1 K can be achieved in commercial white LEDs with this relation. In constant forward current density, Azarifar et al. [87] performed four machine learning regressions including k-nearest neighbor (KNN), radius near neighbors (RNN), random forest (RF), and extreme gradient booster (XGB) on temperature sensitive optical data from over 500 commercial white LED packages and tested the accuracy of prediction with experimental measurements. With near unity in R^2^ scores and small root mean square deviation values, the XGB regressor showed close-to-perfect correlation capability to assess *T_j_* based on SPD behavior. Their recent findings demonstrated that white LED brightness and color characteristics, irrespective of the package’s thermal resistance, can offer a real-time *T_j_* prediction capability.

TSOP-based methods have also been shown to be a practical approach for measuring phosphor temperature in an operating white LED. Based on the total emission division of a white LED to a sum of the spectrum of the blue chip and two spectrums from phosphor with a short and a long wavelength band, Yang et al. [88] examined the fitting peak wavelengths and FWHMs of the short and long wavelength bands at the different phosphor temperatures. They stated that phosphor temperature can be precisely measured by checking the variations of its related emission spectrum. Similar to the LEDs, redshift at higher temperatures was observed. Linear relationships were seen for the FWHM and peak wavelength of the phosphor at different temperatures. However, this model is practical for the same phosphor only, and it cannot be used for mixtures of different phosphor combinations.

**Figure 3:**
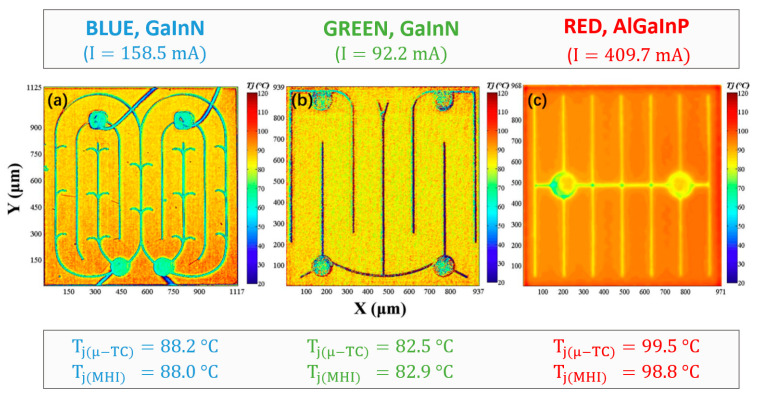
Two-dimensional (2D) temperature distribution of a (**a**) blue, (**b**) green, and (**c**) red LED was measured with microscopic hyperspectral imaging (MHI) under a heat sink temperature of 75 °C. To prevent temperature fluctuations, an average of 2 × 2 pixel temperature was taken in both X and Y directions. Color, material system, and driving current of each LED are given at the top, while *T_j_* obtained by microthermocouple and MHI measurements are given at the bottom. The average standard deviation between T_j(μ-TC)_ and T_j(MIH)_ was found to be 0.9 °C. Reprinted with permission from Ref. [89]. Copyright 2004, IEEE.

Recently, TSOP-based temperature measurement methods have extended to two-dimensional (2D) thermal mapping. Based on new microscopic hyperspectral imaging (MHI), the 2D spectral power distribution can be obtained from light emitting surfaces and can be used to incorporate the TSOP for surface temperature measurement of the LEDs. Jin et al. [89] used the MHI-based centroid wavelength method to study the 2D temperature distribution of the blue, green, and red LEDs (see Figure 3). After calibration of the centroid wavelength coefficient, the authors reached as low as 3 μm resolution for the surface temperature measurement of the LEDs, which is claimed to be capable of reaching submicron level accuracy.

As the next generation micro-LEDs are gaining popularity [90], practices for performance characterization of micro-LEDs are also becoming a topic of interest. Although previous discussions suggest that heat accumulation in micro-LEDs is lower due to enhanced current spreading in active layers with size reduction [91], the studies of Feng et al. [92] and Yu et al. [93] emphasized the importance of *T_j_* for micro-LEDs by stating that the internal series resistance of micro-LEDs is expected to increase with their decreasing size. Therefore, it is important to extend the practical application of TSOP-based temperature measurement to the next generation micro-LED devices. Recently, a thermal study on GaN-based micro-LEDs was conducted by Feng et al. [92]. In their study, a band gap reduction-related redshift of 0.024 eV and FWHM broadening from 27 to 35 nm were seen in the temperature range of 25 to 80 °C. Although they proposed that micro-LED band gap behavior with *T_j_* follows a semi-empirical Varshni relation, it is not possible to determine an accurate coefficient of temperature for the FWHM or band gap for *T_j_* calibration from the presented experimental results.

A parametric summary of TSOP-based experimental LED *T_j_* measurement reports is presented in Table 1.

## 3. Optical Temperature Probing Methods

Optical temperature probing techniques are generally non-destructive and easy to handle with a minimum or no downtime. However, the studied surface should be exposed so that temperature probing can be utilized based on reflection, deflection, interference, fluorescence, birefringence, absorption, or thermal emission optical phenomena [95]. In the case of LEDs, the device cannot be probed with secondary optics due to its determining thermal effect on *T_j_* (refer to Figure 1) [96]. During the measurements, excitation, detection, or the reference light beam is generally adopted, and variations in light properties, including amplitude, phase, polarization, and frequency are recorded for the corresponding temperature evaluation. The employed light beam can be focused/defocused, polarized, and deflected, or operated in modulated and pulsed continuous wave modes to meet the desired measurement objective. Although there are a number of optical temperature probing techniques employed for the thermal characterization of working electronic devices, only infrared (IR) thermography, thermoreflectance, Raman spectroscopy, and liquid crystal thermography were found to be suitable for LEDs and included in this survey (readers can refer to [97,98,99] for further information about excluded optical temperature probing methods). In most of these techniques, measurement signals come from the surface or a region close to the surface of the test LED, which is assumed to assimilate the actual *T_j_*. At the same time, some methods have the potential to provide data from the internal layers of the chip. A critical drawback of some of the optical temperature probing techniques is the possible interference of the active probe source (e.g., focused laser beam) with the device’s electrical and thermal performance, which can be partially avoided by carefully selecting the source’s incident wavelength and power [100,101,102].

### 3.1. Infrared (IR) Thermography

**Figure 4:**
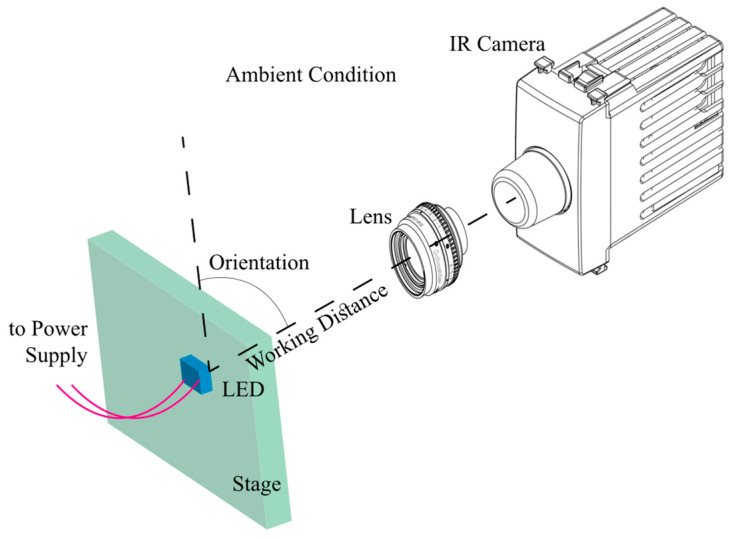
Schematic of the generally used experimental arrangement and apparatus of the IR thermography measurements. Spatial resolution, spectral and sensitivity range, and correct emissivity calibration are all important factors for measurement accuracy.

IR thermal imaging is a real-time imaging method for measuring temperature distributions [103]. The application of IR thermography can be seen for the thermal inspection of various types of LEDs, including chip on board (COB) [104], surface mounted devices (SMD) [105], and flip-chips [106]. This method is widely preferred for various thermal management-related problems in LEDs [107,108,109,110,111,112]. The basic principle of IR thermography is to capture electromagnetic radiation emitted from the surface of objects in the IR spectrum and monitor the corresponding 2D temperature maps. Currently, commercially available IR imaging systems can offer temperature sensitivity within 0.1–1 K and temporal accuracy of 100 μs [113]. Although the IR region of the spectrum extends up to 100 μm, due to the sensitivity reduction above 20 μm, only the 0.7–20 μm range is used for temperature measurements [53]. A typical experimental arrangement and measurement apparatus for IR thermography is illustrated in Figure 4. The setup essentially consists of a lens with a known working distance to focus the thermal radiation on the camera’s detector. Based on the orientation of the LED and ambient conditions, for each setup position, an adjustable emissivity setting should be used to calibrate the detector.

Cheng et al. [114] evaluated the thermal characteristics of red-green-blue (RGB) white LEDs based on thermocouple-calibrated IR thermography, FVM, and the finite element (FE) method with a transparent optical lens still covering the LED chip. Emissivity values of 0.90–0.94 for the red, 0.90–0.93 for the green, and 0.92–0.94 for the blue LED were determined based on the thermocouple calibrations. The probable reason for the significant change in the emissivity was presented as the transparency of the molded lens, IR emission, and reflection from other components of the LED module. Correspondingly, the authors offered a calibration factor to improve the accuracy of the IR thermography measurements. In their analysis, a comparison of the corrected IR measurements and FVM showed an up to 30 °C temperature difference, which reveals that surface temperature and *T_j_* are not equal to each other. Based on the forward voltage verified thermal characterizations conducted by Cengiz et al. [96], a similar emissivity value of 0.9 for the phosphor-converted (pc) LED with a molded lens was found to be an accurate calibration for IR imaging of the chip. In another study, Ozluk et al. [115] studied the effect of the molded lens on the *T_j_* of the green LED by examining thermal and optical properties after the successful removal of the lens from the chip. Based on thermocouple-calibrated results, the emissivity of the green bare LED was evaluated as 0.84. The authors then recommended that proper removal of the chip-covering lens can extend the accuracy and reliability of the IR measurements by decreasing the *T_j_* difference between FVM and IR to 3.9 °C.

**Figure 5:**
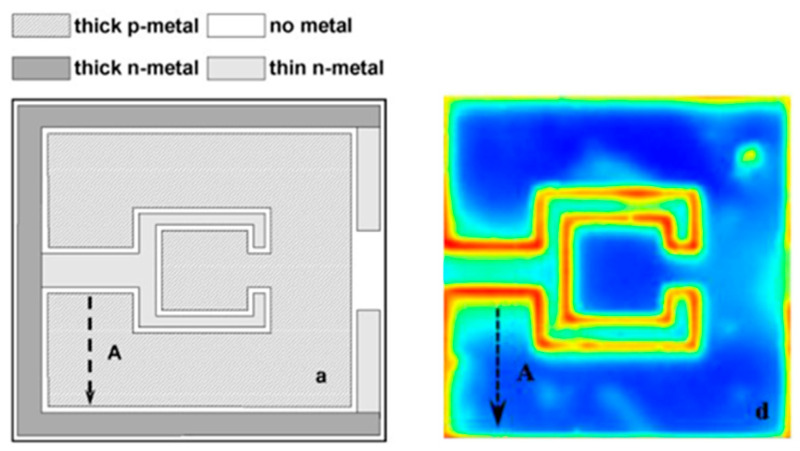
Image of lateral infrared (IR) distribution of a flip-chip LED fabricated with photolithography and dry reactive-ion etching. A schematic of the chip is shown on the left-hand side, while captured IR emissions are shown on the right-hand side. Reprinted with permission from Ref. [116]. Copyright 2013, John Wiley and Sons.

Chernyakov et al. [116] analyzed the thermal distribution in high power flip-chip InGaN/GaN blue LEDs based on IR thermal radiation (see Figure 5). They proposed the use of a 2.5 to 3 µm short wavelength IR camera to improve the spatial resolution of the measurements down to ~3 µm. Moreover, the emissivity variation of each LED part was accounted for with temperature-controlled preliminary calibrations, which were claimed to provide less than 2 K temperature accuracy for IR measurements. Likewise, Wu et al. [66] conducted temperature-controlled calibrations to determine the emissivity of materials of a high power multi-chip COB LED. Following the successful calibration of the IR camera, the difference between measurement and numerical simulation was found to be less than 2%. Chang et al. [107] preferred to calibrate the emissivity of the GaN-based green LED by black painting the empty surface of the LED wafer as a reference point. As a result of the applied correction algorithm for the adjustment of response and superimposed offset signal images, the reflected radiation was analyzed, and the emissivity was corrected for each pixel. In a more recent study, Aladov et al. [117] suggested the use of a film coating with transparency in the visible region and absorption in the IR region. Application of a special thin-film coating aims to prevent emission absorption of the LED under test conditions and ensure real temperature mapping without falsification. A comparison of the captured IR images with and without coating is given in Figure 5.

Diffraction-limited spatial resolution and local temperature uncertainties are two major issues of IR thermography measurements [118,119]. Even though diffraction-limited spatial resolution is an inherent drawback, local temperature uncertainties can be dealt with by the calibration of the changing radiative properties of the inspected surfaces. With that being said, accurate determination of the emissivity may be problematic due to significant variation in the radiative properties of the materials in the heterostructure and packaging. Semiconductor layers, metallic electrodes, interconnections, coatings, and bonding elements have differing transparency or reflectance to infrared radiation, which results in the wrong interpretation of collected radiation and interference of emissivity values of the sublayers [119,120]. To overcome this issue, researchers have been employing various calibration techniques such as two temperature maps [121,122], irradiance based emissivity corrections [123], high emissivity coatings [124], and radiative micro carbon particles [125], for the correct determination of the surface emissivity.

In general, thermal concerns still exist on the emissivity of LEDs which requires another source of reliable temperature measurement technique, usage of supplementary black coating or microparticles that may cause heat spreading, damage, or visible emission absorption that disturbs the operational thermal and optical behavior of the LED. As depicted in Table 2, the material emissivity of semiconductors can vary significantly at different spectral bands, and for interconnection metals, much lower emissivity values can be observed [126]. In addition, parameters such as humidity and ambient and atmospheric temperatures near the inspected object also have an unignorable influence over the radiation properties [103]. Semiconductor doping also modifies the radiative properties of the active region, and as shown in the study of Welna et al. [127], when free electron concentration is increased up to 10^−19^ cm^−3^ in the n-GaN active region, shifting towards shorter wavelengths (from 7 µm to 2 µm) occurs in the infrared absorption band. One of the latest studies conducted by Rongier et al. [128] thoroughly discussed these property changes for different spectral bands and opacity regions. By taking the emissivity variations into account, the authors successfully developed an in-lab IR thermography test bench that is calibrated for quantitative thermal analysis of high luminance LED front lights. A parametric summary of IR thermography-based experimental LED temperature measurement reports is presented in Table 2.

### 3.2. Thermoreflectance

**Figure 6:**
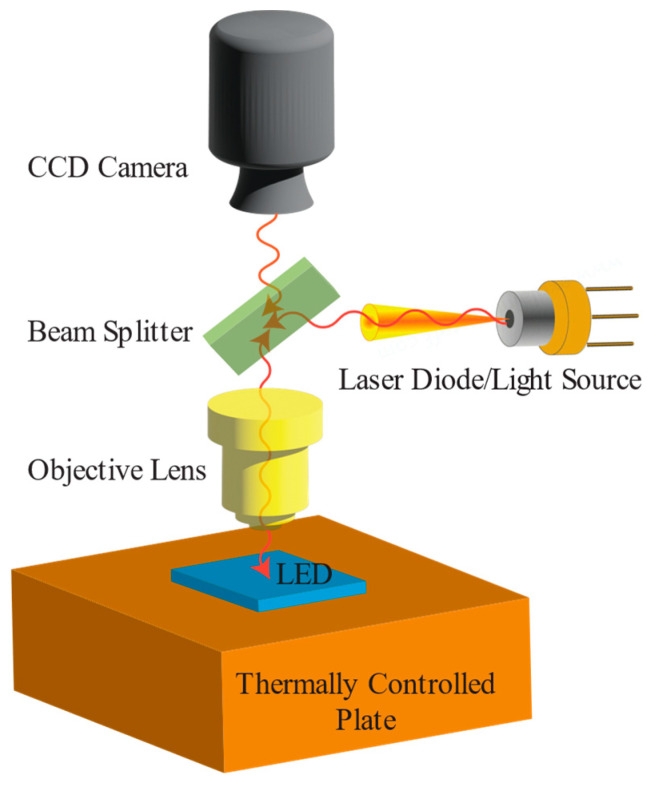
Schematic of a typical measurement arrangement and experimental apparatus used for the thermoreflectance setup.

Thermoreflectance microscopy is a high resolution and non-contact thermal profiling method that measures the relative change in the reflectivity over a surface and maps the temperature distribution according to the reflectivity variation [130]. Even though the temperature dependency of material reflectivity is usually in the order of 10^−5^ to 10^−4^ K^−1^ [131], temperature-induced changes can be detected by incorporating a sensitive (amplified) measurement system. The benefit of thermoreflectance imaging is in its ultrafast temporal resolution of up to 800 ps and spatial resolution of 200–250 nm if UV or visible illumination is used [132]. Moreover, it is possible to optimize the thermoreflectance for a wide range of materials, and it has been used to measure the temperature of various electronic components [133,134,135,136,137,138,139]. A typical measurement setup for the thermoreflectance method is demonstrated in Figure 6 as a reference.

The temperature resolution of the thermoreflectance method depends on the noise generated during measurements and the detector’s sensitivity, while the wavelength of the optical source limits the spectral resolution. In some cases, reflected and emitted light from beneath the LED surface can interfere with the surface reflectivity of the chip and can lead to an inaccurate surface temperature measurement result. To avoid this issue, Summers et al. [140] employed thermoreflectance imaging using a confocal microscope to measure the surface temperature of the AlInGaP red LED. Results from widefield and confocal imaging were compared and verified with the FVM. A negative offset in the widefield temperature measurement method was observed, and the advantage of confocal microscope imaging was shown. In another study, Ling and Tay [141] used the thermoreflectance thermography method to study the surface temperature of phosphor-coated blue LEDs. Measurements were carried out with a 5X zoom microscopic lens with a light source of 405 nm wavelength, claiming to have the lowest emission from the chip itself. The LED surface reflectivity variation with temperature was calibrated, and a relative reflectivity coefficient of 1.71 × 10^−5^ 1/K was determined.

Xiao et al. [142] proposed using the relative reflected intensity of the incident exciting light to determine *T_j_*. The lock-in method was used to extract the interference of the emitting light from the LEDs and increase the measurements’ dynamic range. Their proposed technique was calibrated with micro thermocouple measurements. They stated concerns about the calibration using thermocouples due to the difference in diameters between the exciting light spot and the thermocouple and the uniformity in thermal distribution on the surface of the chip.

Kendig et al. [143] made use of thermoreflectance imaging to determine the 2D temperature maps of encapsulated UV and blue LEDs. Thermoreflectance signals were obtained with a custom 1-megapixel charge-coupled device (CCD) system, and the lock-in technique was implemented to increase the measurement resolution. The wavelength, surface roughness, and material-dependent thermoreflectance coefficients were calibrated to measure quantitative temperature values. As a result, the thermoreflectance method was found to be adequate for investigating thermal non-uniformities and the transient thermal responses of the LED.

In one of the latest studies, the reflective light intensity change of a blue GaN LED was exploited to derive the 2D transient temperature distribution of the operating device [144]. In contrast to other studies, the authors captured light reflection with a high-speed camera with a 505 μs to 68 μs time resolution range. To prevent a band gap modulation effect, a red LED with a 690 nm wavelength was employed as an illumination source. According to the authors, the proposed measurement method provided better spatial resolution than traditional optical probing methods, with the degradation from the homogeneity of the 2D temperature distribution occurring only at high frame rates due to the shorter exposure time.

**Figure 7:**
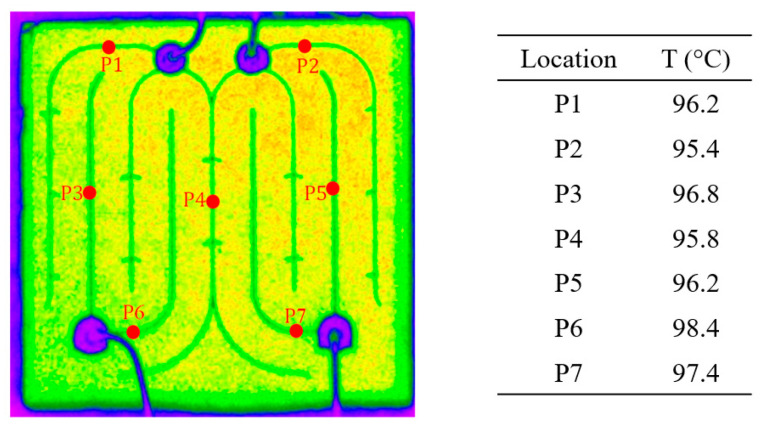
The surface temperatures of a blue LED were measured at different locations with the thermoreflectance method. Reprinted with permission from Ref. [145]. Copyright 2018, American Physical Society.

Recently, Zheng et al. [145] employed the thermoreflectance method to study the surface temperature of lateral-type blue LED chips (see Figure 7). In their measurements, a reflectivity change of the golden metal electrode was recorded, and it was observed that the thickness of the electrode was sufficiently thick to hide unwanted reflections from the underlying materials. For the thermoreflectance experiments, an incident probe laser beam with a spot surface of 3 μm was used. As a result, good agreement between the thermocouple, FVM, and thermoreflectance methods was observed in the *T_j_* range of 75 °C. However, discrepancies between the different methods were seen at higher temperatures, where FVM showed obvious higher values. The authors stated that this difference is due to the self-heating and finite thermal resistance between the *T_j_* and measured surface points. It should be mentioned that as *T_j_* increases, significant heat flux from the surface to the ambient due to a higher transfer coefficient can be expected [146], which can result in the underestimation of the surface thermography of LEDs.

### 3.3. Raman Spectroscopy

**Figure 8:**
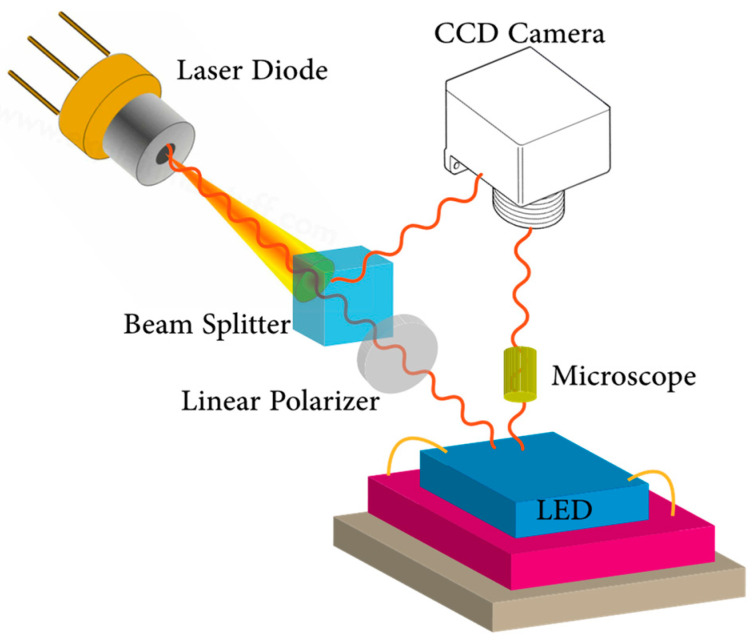
A schematic of a typical measurement arrangement and experimental apparatus used for the Raman spectroscopy setup.

Raman spectroscopy is a non-contact temperature mapping technique that can provide submicron spatial resolutions up to 1 μm [147] and nanosecond temporal resolutions up to 200 ns [148] during time-resolved measurements. With this technique, temperature distributions across the active region of semiconductors formed by Raman active material layers are evaluated based on their phonon frequency. A typical Raman system is composed of an excitation source (often a laser) that is focused on the LED, a beam splitter, a sample holder, and a spectrometer that can detect the frequency shifts (see Figure 8). As an excitation source, a variety of lasers, such as argon ion (488.0 and 514.5 nm), krypton ion (530.9 and 647.1 nm), He:Ne (632.8), Nd:Yag (1064 and 532 nm), and diode laser (630 and 780 nm) can be employed [149]. Temperature measurement errors due to excitation source-induced heating and carrier generation can be reduced by lowering the source or by choosing a source with a wavelength below the band gap of the LED (see Table 3).

It is worth mentioning that Raman spectroscopy temperature measurement of large surface areas may be impractical due to the need for raster scanning and data integration [119]. Moreover, fine temperature profiling is time consuming because detecting weak Raman signals might not be easy [126]. Nonetheless, the Raman spectroscopy approach is capable of capturing temperature profiles of micron size features with high spatial resolution and it has been studied by many researchers for thermal characterization purposes (e.g Diamond [150], AlGaN/GaN [151,152,153], Graphene [154], and others [155,156,157,158,159]).

In an early study conducted by Schwegler et al. [156], the *T_j_* of InGaN-based LEDs was obtained using micro-Raman spectroscopy. The device was driven with moderate level input currents, up to 30 mA, with a power density of 705 W/cm^2^. Temperature evaluations were made considering the shift in GaN E_2_ (high) phonon frequency with temperature, and in order to reach high spatial resolutions, the authors combined a 514 nm wavelength laser excitation source with a 0.7 μm spot diameter microscope lens. The results from the micro-Raman spectroscopy, TSOP, and FE models were found to be in good agreement, and *T_j_* was evaluated to be 140 °C for 30 mA input current. In another study, Chitnis et al. [155] used micro-Raman spectroscopy mapping to evaluate the *T_j_* of a 325 nm flip-chip deep UV LED. Similar to the previous analysis, the E_2_ (high) phonon frequency shift of AlGaN was examined, and to prevent light absorption by the device, a 488 nm wavelength laser that had below band gap excitation was used as a source. The LED was driven with low input currents up to 50 mA and the highest surface temperature was determined to be 70 °C, indicating a good agreement between TSOP and micro-Raman spectroscopy results. In a later study, Senawiratne et al. [160] presented a *T_j_* analysis of GaN-based blue and green LEDs with sapphire and GaN substrates by micro-Raman spectroscopy. Measurements were conducted with a 785 nm wavelength laser excitation source while the Raman frequency shift of E_2_ (high) phonon was investigated. Different from previous studies, the LEDs were driven with relatively higher input currents, up to 250 mA (357 A/cm^2^), and Raman peak shifts to the lower wavenumber sides were observed due to thermal lattice expansion at high current levels. Considering the spectral resolution of the spectrometer used for the measurements, the accuracy of the micro-Raman technique was evaluated to be ±7 °C.

**Figure 9:**
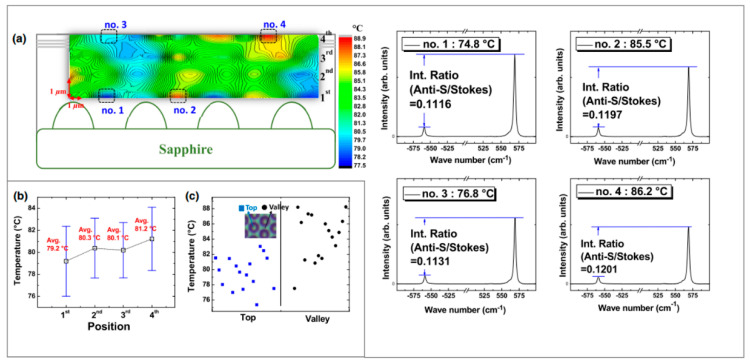
(**a**) Temperature distribution in the x-z cross-section obtained from Stokes and anti-Stokes intensity ratio evaluations. Extreme Raman spectra points are shown from no: 1-4. LED *T_j_* is assumed to be the same as the top surface temperature. (**b**) Average temperatures at different locations. (**c**) Scatter temperature plot of 32 different positions at top and valley sites. Reprinted/adapted with permission from Ref. [163]. Copyright 2018, American Physical Society.

It should be noted that both thermal and stress fields have a strong influence on the Raman frequency shifts. This means that the phonon shifts captured by the spectrometer may be induced by simultaneous effects of both temperature and stresses under combined thermomechanical loadings. Therefore, to reach high accuracy results, distinction of thermal strain effects should be made for the linewidth of the Stokes shifted peak [161]. Wang et al. [162] acknowledged this issue for the *T_j_* evaluation of an unpackaged UV LED. In their analysis, a 442 nm wavelength laser with a 5 μm spot size was used as an excitation source, and the spectrometer’s integration time was reported to be 3 s. In order to evaluate the stress effects over the peaks, the Raman spectrum of a stress-free bulk GaN wafer was also analyzed. Their findings showed that the E_2_ (high) Raman peak position of the LED chip was 1.8 cm^−1^ higher than the stress-free GaN wafer. This upshift was mainly attributed to compressive stresses of the GaN layer grown on sapphire. To overcome this issue, the Raman spectroscopy findings were corrected for stress-induced shifts. Following that, FWHM and the position of the Raman peak were determined by curve fitting with a Lorentzian function. Compared to the FWHM, evaluation of the Raman peak position was found to be less stress-dependent and to provide higher accuracy. More recently, Park et al. [163] performed a confocal micro-Raman microscopy-based temperature measurement of GaN-based LEDs and achieved approximately 1 μm diffraction-limited spatial resolution and ±2 °C accuracy. Instead of Raman frequency shift, the authors evaluated the anti-Stocks and Stokes Raman intensity ratio change unaffected by the heating-induced stress relaxation. (see Figure 9).

Recently, Tamdogan et al. [164] used the Raman spectroscopy method to evaluate the junction temperature of GaN-based blue LEDs with and without phosphor coating. Experimental measurements were handled using three different laser excitation sources (532, 440, and 325 nm) with a 1 μm beam diameter and 50X objective. For the measurements, bare and coated LEDs were driven with 150, 300, and 400 mA input currents, and the Raman spectroscopy findings were compared to the IR and FVM analysis for validation. Even though the measurement results for the uncoated LEDs were found to be concurrent with the other measurement methods, bare-chip and phosphor-layer Raman emissions were overlapped for the coated LED, which, in fact, limits the usage of the Raman spectroscopy for the phosphor-coated LED chips. One way to overcome this issue was found to be the use of Raman responsive microparticles. This is a common practice to broaden the applicability of the technique in which Raman active particles such as anatase TiO_2_ are embedded in an LED to capture temperature gradients across the surface [165,166]. The application of nanoparticles can also provide higher spatial resolution for probing temperatures of small-length scale features [167]. However, due to thermal resistance between the surface and the p-n active region, TiO_2_ particles over the LED surface only give insight into the surface temperature and are not sufficient to determine the actual *T_j_* of the LED.

**Figure 10:**
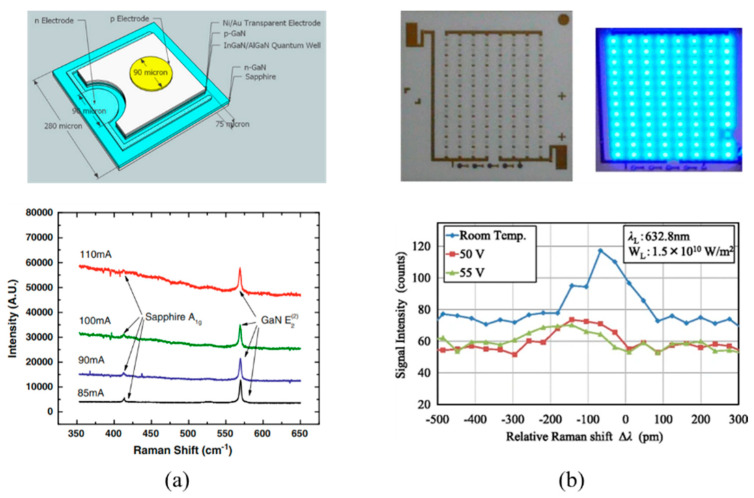
(**a**) Junction temperature determination of UV LED with micro-Raman spectroscopy. The structure of the UV LED chip is shown in the upper figure, while the E_2_^(2)^ Raman peaks at different input currents are shown at the bottom. Reprinted with permission from Ref. [162]. Copyright 2010, Springer Nature. (**b**) Junction temperature estimation of a blue LED using pulsed laser Raman scattering. Measured LED package is shown in the upper figure, while a Raman spectra of the E_2_ at different input voltages is shown at the bottom Reprinted/adapted with permission from Ref. [168]. Copyright 2015, Springer Nature.

Literature examples for the usage of Raman Spectroscopy for *T_j_* unpackaged UV LED and blue LED are given in Figure 10a,b while a parametric summary of Raman Spectroscopy studies is provided in Table 3.

### 3.4. Liquid Crystal Thermography

**Figure 11:**
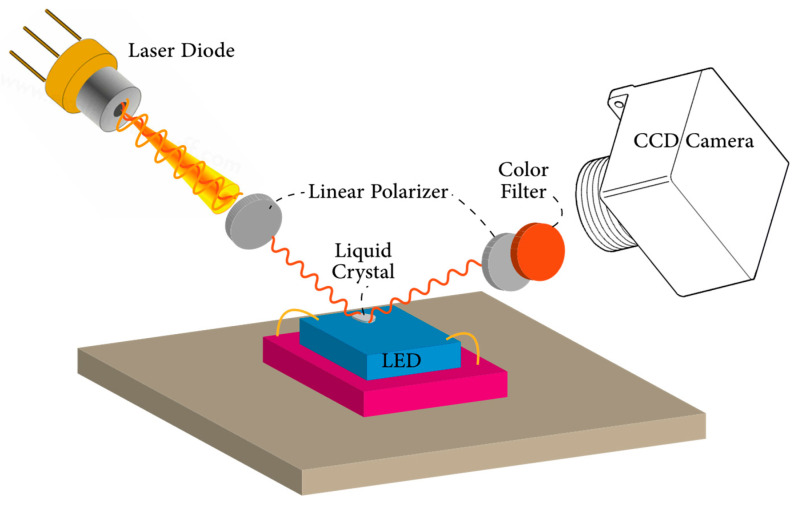
A schematic of the experimental arrangement of the liquid crystal thermography technique. The test setup is typically composed of a polarized laser beam, a charge-coupled camera with a color filter, and liquid crystal coated over the LED surface.

During the phase change of liquid crystals and at the intermediate phases where both liquid and solid molecular structures exist [170], light reflection shows distinct behaviors at specific temperatures [171]. Considering this temperature sensitivity, liquid crystals can be applied over the surface of the device, and reflections at unique wavelengths and colors can be used to assess surface temperatures [172]. The selection of suitable liquid crystal compounds is determined by the transition temperature between phases. Commercially available liquid crystals enable temperature profiling between the range of 30 to 120 °C with 0.5 to 30 °C increments [173,174]. For reference, a typical experimental measurement arrangement of the liquid crystal thermography method is depicted in Figure 11.

At temperatures below the transition point, liquid crystal will be optically anisotropic, causing the polarization orientation of the reflected light to shift. Therefore, areas with temperatures below the transition point appear bright under a microscope/camera. On the contrary, if the temperature of the liquid crystal rises above the transition point, it will reach a complete isotropic fluid phase. Correspondingly, the polarization orientation of the reflected light will not change, and areas with temperatures above the transient point will seem black. Furthermore, at the boundaries between the black and bright regions, the temperature of the liquid crystal will be the same as its transition temperature. This means that by knowing the exact transition temperature of the liquid crystal, it is possible to assess the temperature distribution across the LED device based on the visual appearances.

**Figure 12:**
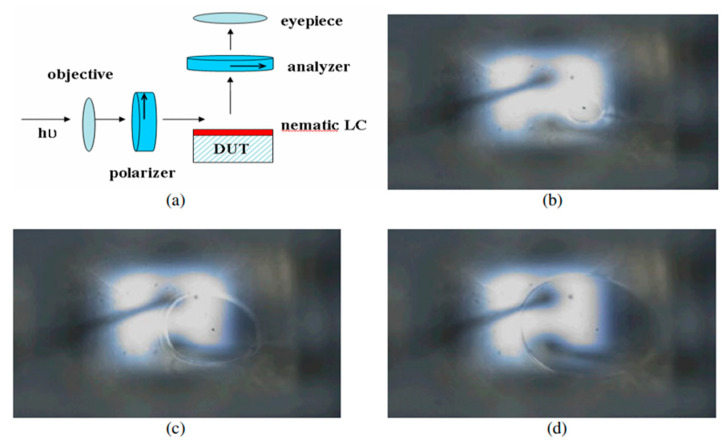
(**a**) Schematic setup of liquid crystal thermography measurement of GaN based blue LED. (**b**–**d**) Micrographs of dark gray hot spots that are above the liquid crystal transition temperature. The hot spot size increases with input power. Reprinted with permission from Ref. [176]. Copyright 2004, John Wiley and Sons.

Lee and Park [175] were among the first to use nematic crystal thermography for temperature measurement of visible LEDs. Liquid crystals with transition temperatures of 302, 313, 331, 356, and 380 K were applied over the LED chip, and a red filter was used to reduce the LED’s optical power, which can otherwise overwhelm the reflected light from the chip. A high power 660 nm laser beam was used as the illumination source since the chip is transparent to this wavelength, and it was ensured that the laser beam did not heat up the device. Due to the blockage of LED light, the authors suggested the use of a transparent high-power laser for accurate spatial temperature measurements. Although the black and bright appearance of the liquid crystal was used to assess the temperature of the LED device, and the authors were able to reach 21 and 35 μm resolutions, measurements were limited by the transition temperatures of the liquid crystals. In a similar procedure, Hwang et al. [176] used the liquid crystal thermography technique to observe local hot spot zones in GaN-based blue LED chips with a size of 330 × 330 µm^2^. For the measurements, a liquid crystal with a 29 °C anisotropic–isotropic transition temperature was used, and localized hot spots of 80 and 400 µm size were observed (See Figure 12).

The spatial resolution of liquid crystal thermography can be extended from 2 to 4 μm and can be less expensive than other optical probing methods; however, the uniformity and thickness of the liquid crystal can affect its accuracy and resolution [97]. Therefore, temperature errors caused by heat spreading and temperature profile distortion should be taken into account during the measurements. Although high spatial resolutions can be reached with this technique, it can only detect local hot spots over the LED surface but not the actual *T_j_*. It can be seen that due to the complexity of the experimental setup, coating difficulties, and uncertainty of its thermal effects, liquid crystal thermography is not a common LED junction temperature measurement technique.

## 4. Temperature Sensitive Electrical Parameters (Tseps)

Similar to the TSOP-based methods, semiconductor *T_j_* measurements can also be performed via exploiting electrical sensitive parameters (TSEPs) of the device under study. Typically, temperature measurements with TSEPs are comprised of two stages. First, the TSEP response of the device is calibrated at different temperatures. To perform the calibrations, an external system such as an oven, a dielectric bath, or a thermally controlled hot plate is generally employed to heat the device while the change in TSEP is measured. Then, in the latter stage, the relationship formed in the calibration phase is utilized to determine the actual *T_j_* at operational current levels. The main advantage of the TSEP-based methods is that measurements can be made on fully packaged devices without a need to remove any package component for visual or mechanical access to the chip, Ref. [54] or in most cases, they require no addition or modification to the device’s configuration. However, the spatial resolution of TSEPs relies on the spatial distribution of the TSEPs characteristic of the device, and, mainly, the average temperature within the active region is being evaluated. In addition, non-isothermal distributions within the device may introduce additional errors due to averaged measurement values. Forward voltage, threshold voltage, leakage current, gain, and resistance are the most common TSEPs used to measure the temperature of semiconductor devices. Since the forward voltage (*V_f_*) is the most common and preferred technique to determine the *T_j_* of LEDs, it has been reviewed individually in the upcoming *Forward Voltage Method (FVM)* section. Compared to the FVM, there are only a few other TSEP-based temperature measurement methods in the literature specifically employed for LEDs, and they are discussed concurrently under the section named *Other TSEP-based Methods*.

### 4.1. Forward Voltage Method (FVM)

LED product manufacturers and researchers generally prefer the well-known FVM for *T_j_* estimation of LEDs. Whether it is phosphor-converted LEDs [164], multi-chip configuration [177], or an LED lamp [178], FVM has been shown to be a promising approach to estimate the temperature of the active region. Therefore, a number of examples of FVM for *T_j_* evaluation of LED devices can be found [179,180,181]. Notably, the claimed accuracy of this method ranges from ±3 [62,82] to ±0.88 °C [182].

**Figure 13:**
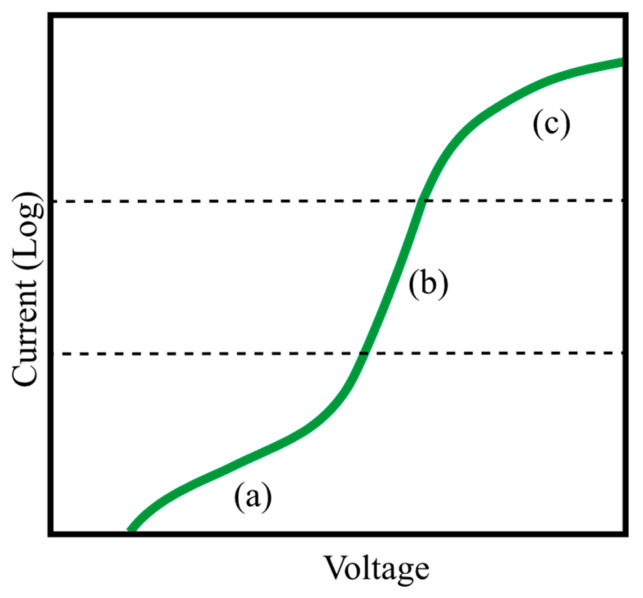
Typical semilog forward voltage behavior of an LED.

FVM exploits the temperature dependence of the forward biased diode’s voltage by passing a constant current through the LED and monitoring voltage flow across the diode. A typical forward current-voltage characteristic of an LED is represented in Figure 13. The plotted current–voltage curve is comprised of three regions. The low-current section (see region (a) in Figure 13) represents the region where current–voltage behavior is dominated by the trap-assisted tunneling or defect-assisted carrier leakages (Perlin et al. [183] broadly discussed the dominancy of carrier transport by tunneling across the active region, and readers can refer to their study for more information about the topic). As the voltage increases, an exponential increase in current can be observed (see region (b) in Figure 13), which is dominated by radiative recombination. Finally, when the voltage exceeds a certain limit (e.g., 2.5 V for blue LEDs), deviation from exponential behavior is observed (see region (c) in Figure 13) due to the increased role of ohmic series resistance.

Exponential behavior is observed in the region (b) in Figure 13, where a small increase in the voltage results in a significant current flow in the diode, which brings the opportunity of recording voltage values that can change with temperature with very small pulse currents (to avoid thermal perturbation). Within the exponential current interval, researchers are finding a linear relationship between *V_f_* and *T_j_* with a slope that is dependent on the drive current. In the study of Keppens et al. [184], the theoretical basis of the linear behavior was well-discussed. As diffusion and space charge recombination current mechanisms can be observed in LEDs, experimental *I_f_*–*V_f_* characteristics can be modeled by the experimental Shockley equation [184]:

(1) $$I_{f}=I_{s}e^{\left(\frac{eV_{f}}{nkT_{j}}\right)}$$

where *I_s_* is the effective saturation current (combination of recombination saturation and diffusion saturation currents), and *e*, *n*, and *k* stand for the elementary charge, ideality factor of the diode (theoretical value between 1 to 2), and the Boltzmann constant. Furthermore, *I_s_* can be modeled as:

(2) $$\ln\left(I_{s}\right)\approx \frac{\left(\frac{2E_{a}}{nk}\right)}{T_{j}}+\left(\frac{\alpha }{nk}+\ln\left(C\right)\right)$$

where *α* is a positive constant, *E_a_* is the activation energy approximated by the Varshni formula in a temperature range of 300–400 K [60], and *C* is a quasi-constant factor. By combining the two equations, forward voltage can be written as:

(3) $$V_{f}\approx \left[\frac{nk}{e}\ln\left(I_{f}\right)-\frac{\alpha }{e}-\frac{nk}{e}\ln\left(C\right)\right]T_{j}+\frac{2E_{a}}{e}$$

which shows a constant temperature coefficient of voltage depending on the forward current of the LED.

In some cases, due to faults in connections to the p-n junction, higher series resistance, or low quality of the heterostructure manufacturing, the current interval in the exponential region cannot be achieved. This issue can block the possibility of calibration with low currents or achieving a meaningful relationship from calibration. Typically, the calibration process should involve short periods of pulses since heat can still be generated when the device is electrically active to non-radiative recombination, radiation absorption, and Joule’s heating in an LED [15]. Therefore, applied pulse values and durations should be selected such that heat generation in this period does not interfere with the calibration process [63].

In one of the earliest attempts, Xi and Schubert [185] developed an expression for the *T_j_*–*V_f_* relation and showed that constant values for the temperature coefficient of −2.3 mV/K at a temperature range of 20–120 °C exist for the studied GaN-based UV LED. This is known to be the first study that investigated the dependence of junction temperature on operating voltage for GaN LEDs grown on a sapphire substrate. Furthermore, Jiang et al. [186] were the first to report the *T_j_* characteristics of GaN-based blue LEDs on Si substrate. In their study, a constant temperature coefficient of −3.0 mV/K at a temperature range of 30–90 °C was recorded. When compared to LEDs on sapphire, the *T_j_* was found to be much lower, which was mainly attributed to the thermal conductivity difference of Si and sapphire. Meyaard et al. [187] looked at the temperature coefficient of GaInN LEDs from 80 K to 450 K and found two-slope characteristics for *T_j_*–*V_f_* temperature coefficient of −8 mV/K from 80 K to 100 K and −1.7 mV/K from 200 K to 450 K.

In recent studies, it was claimed that the constant temperature coefficient voltage assumption for FVM is not always accurate. Onwukaeme et al. [188] investigated the *T_j_* of GaN-based blue LEDs using the non-linear dependence of *V_f_* at temperature ranges of 20–100 °C. In their experiments, 100 mA pulses were used in the calibration process, and quadratic fitting was claimed to be more accurate than linear fitting for the temperature coefficient of voltage. The deviation reported in this research can also be seen in other studies where relatively high pulse values were used in the calibration process [189]. For instance, Kim and Han [190] investigated the *T_j_* dependency on the heat dissipation of a GaN-based blue LED by analyzing the decrease in *V_f_* at elevated temperatures. FVM was employed to determine the *T_j_*, and the LED was calibrated by applying a 5 mA current for 10 ms. In their analysis, deviation from the linear behavior was observed above 125 °C, and a cubic polynomial was found to be the ideal fit to characterize the relation at high temperatures. Linear deviations in high pulse values can be attributed to junction heating that leads to calibration of the *V_f_* to a higher *T_j_*. At high temperatures, thermal droops also increase in the LEDs, which, indeed, intensify the self-heating of the LED [191,192,193,194]. At low temperatures, deviation from the constant voltage coefficient of temperature can be explained by internal series resistance [184]. However, at low pulse durations, which ensures no *T_j_* rise [63] and falls into the exponential current interval (e.g., 1 mA), numerous perfectly linear *T_j_*–*V_f_* were reported in the literature [64,82,94,96,115,129,164,182,195,196].

With the rapid growth of LED products, FVM-based commercially available test devices for their thermal investigation have become available. One well-known measurement equipment that utilizes FVM is the commercially available transient thermal T3ster [197]. Measurement with T3ster equipment comprises both hardware measurements and software calculations in compliance with the JEDEC standards [198,199,200]. The equipment can record the dynamic temperature responses with submicron time resolution via single or multi-port measurements. In addition to the *T_j_* evaluations with respect to the *V_f_* drop across the p-n junction region, the heat flow path from the device to the ambient and thermal resistances can be measured.

Yang et al. [201] used the T3ster measurement system to determine the *T_j_* and thermal resistance of a PAR 38 light bulb. In their analysis, k-factor (temperature coefficient of voltage) calibrations were made when the LED was driven with 1 mA bias current, and the ambient temperature was changed from 25 to 85 °C. Correspondingly, a nearly linear *V_f_*–*T_j_* relation was realized with a 5.97 K/V calibrated k-factor, and further thermal characterizations were made for a 36 mA operational current. In another study by Yang et al. [202], thermal resistance measurement of an LED module was made using T3ster. In the experiments, the LED was driven with 3.2 W input power and the overall thermal resistance of the module was found to be 4.10 and 9.18 °C/W for two different heat pipe substrates. Based on the instrument specifications and measurement conditions, the uncertainty of the thermal resistance measurement with the T3ster system was found to be 11.3%. Liu et al. [203] used T3ster combined with an integrating sphere to evaluate the *T_j_* and thermal resistance of a blue LED mounted on an aluminum plate. For the measurements, the time of the test system delay was set to 1 μs with a temperature measurement accuracy of 0.01 °C. A linear relationship between *V_f_*–*T_f_* was formed with a value of −1.043 mV/°C. The LED was driven with a 300 mA of input current for 60 s at 25 °C ambient temperature while the sensor current was 10 mA for 100 s. As a result, T3ster *T_j_* calculations and simulated *T_j_* values showed similar trends with a nearly 1 °C difference. Yang et al. [204] measured the *T_j_* and thermal resistance of an organic light emitting diode (OLED) using T3ster. During the k-factor calibrations, the OLED was driven with a 1 mA sensor current at a temperature range between 15 and 55 °C with a 5 °C temperature interval. A linear relation between *V_f_* and *T_j_* was verified with a k-factor of −0.023 mV/°C. According to their thermal characterizations, the thermal gradient between *T_j_* and the case temperature was found to reach up to 14.5 °C when the OLED was driven with a 120 mA input current with an optical efficacy of 18.3 lm/W. Further examples of using T3ster can be found in a number of studies [85,105,111,205,206,207,208].

In JEDEC JESD51-51 [198], it was stated that to perform k-factor calibration, an isothermal environment is required. Concerns exist about the accuracy of the thermal equilibrium of the commercially available temperature measurement systems during the k-factor calibration process. Typically, to achieve thermal equilibrium and to obtain the corresponding *V_f_*, LEDs soldered on a PCB are mounted on a thermally-controlled heat sink. Then, the heat sink temperature was set to a predefined measurement value. In these configurations, non-isothermal environmental conditions or thermal resistance differences between the thermocouple and LED may cause calibration inaccuracy [28]. This concern was also recently pointed out by Hantos et al. [189] in the framework of the Dephi4LED project [209]. As mentioned by the authors, when attachment on temperature-controlled heat sinks is used, heat transfer toward the air builds up a parallel heat-flow path which disturbs the accuracy of the k-factor calibration, and the same issue exists in the test phase.

The arrangement of suitable thermal environments and the selection of appropriate electrical inputs are crucial steps in conducting accurate FVM measurement experiments which are discussed in [210]. During the conventional FVM calibration process, the desired “n” pulse temperature (T_p,(1, 2, …, n)_) should be kept constant for several minutes to perform “m” number of pulses (P_1, 2, …, m_) with a control interval in between each pulse (to). Oven insulation and active heater controls are commonly used to maintain equilibrium conditions for measurement ranges. Even though the isothermal condition can be more accurate at temperatures near to the room temperature, heat dissipation from the oven and air motion inside the oven increases temperature fluctuations, and thermal resistance between the calibration thermocouples and *T_j_* increases. To ensure pulse current temperature is equal to the *T_j_*, an oven arrangement as configured in the EVAtherm system can be used [29,96,115,129,181,210] in which LEDs are placed in the middle of a thermally-controlled oven with no direct contact between the heater-embedded walls. In this configuration, several thermocouples record the air temperature and PCB to increase the speed and accuracy of the isothermal condition. In addition, fins can be used to suppress the air motion and increase thermal uniformity. Thus, thermal resistance differences and radiation heat transfer issues can be controlled, and the *T_j_* at the desired temperature can be achieved with more assurance.

**Figure 14:**
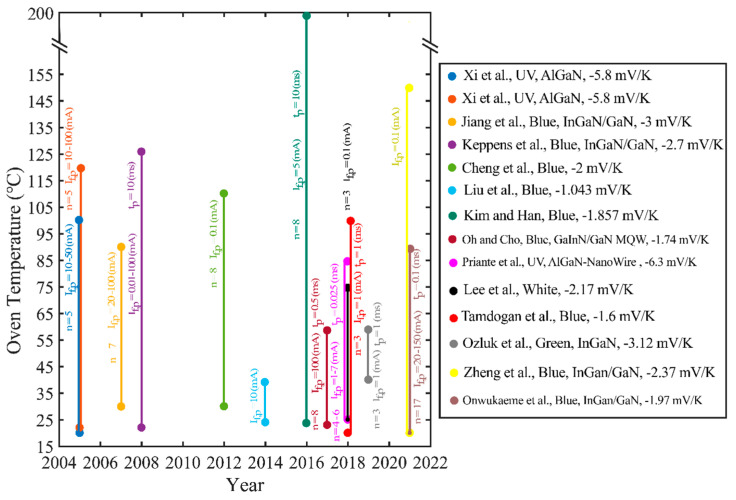
A yearly summary of the employed oven temperature ranges for the calibration of FVM from different research groups. I_f,p_ represents the applied pulse current, t_p_ represents the duration of the applied pulse current, and n stands for the number of temperature points set for the calibration measurement within the given oven temperature interval. The legend provides author indications (oldest to most recent from top to bottom), studied device types, and k-factors obtained from the pulses with a minimum value of I_f,p_. Each colored line indicates the temperature difference between T_p,1_ and T_p,n_. References from top to bottom are Xi et al. [62], Xi et al. [64], Jian et al. [186], Keppens et al. [184], Cheng et al. [114], Liu et al. [203], Kim and Han [190], Oh and Cho [196], Prinate et al. [94], Lee et al. [111], Tamdogan et al. [164], Ozluk at al. [115], Zheng et al. [145], and Onwukaeme et al. [188]. Reprinted with permission from Ref. [210]. Copyright 2022, Institute of Physics and the Physical Society.

Figure 14 compares experimental parameters from different research groups that performed FVM calibrations in a concise manner. As shown in Figure 14, a wide range of pulse current (I_f,n_) values were reported, while only a few authors provided pulse duration (t_p,n_) values. It can be seen that, in general, only a few pulses are performed, and the value of “n” rarely exceeds 10. This issue arises from hardship and the time-consuming process of achieving a thermal equilibrium condition. Initial temperatures are mostly adapted near the room temperature; however, consistency in the value of maximum temperature cannot be seen. Interestingly, these inconsistencies in experimental parameters have shown their effect on the behavior of voltage–temperature, which can be seen in more detail in Table 4, where experimental measurement parameters employed in various FVM-based LED thermal characterization studies are summarized.

### 4.2. Other TSEP-Based Methods

Despite the fact that FVM can be used by an arrangement to measure the *T_j_* of AC LEDs [212], Zhu et al. [213] introduced a new method for AC LED *T_j_* measurement. In this method, a periodic bipolar voltage pulse signal was applied as an input while the amplitude of the output current was calibrated as a temperature sensitive parameter. The accuracy of the measurement was tested via direct thermocouple chip measurement, and a 1.2% relative deviation was observed between these measurement methods. Other TSEP methods, such as the threshold voltage method for AC LEDs [214] and low forward current method [215] can also be found in the literature. For instance, Zhao et al. [216] determined the resistance of n-GaN layer as a function of temperature and used this relation to evaluate the *T_j_* of a blue LED. The electrodes of the chip were attached to the resistance meter with ohmic type connectors, and the chip was calibrated with 5 mA for 0.5 s pulse currents and 20–195 °C ambient temperatures to form the resistance vs. *T_j_* relation. Experiments were repeated for five LED dies from the same batch to ensure the consistency of the measured data and an interval shift of 0.1 Ω for separation of the mixing curves. The accuracy of the method was claimed to change between ±3 and ±1 °C for *T_j_*, increasing from 30 to 120 °C. Validation of the temperature-dependent resistance method with FVM showed that the proposed technique is a promising approach for the *T_j_* measurement of LEDs. Wu et al. [217] proposed an interesting approach to avoid heating during pulse current calibration of TSEPs. Instead of forward voltage, the authors suggested the use of temperature-dependent reverse current to determine the *T_j_* of InGaN-based blue LEDs. In addition to the prevention of excess heating in pulsed conditions, the authors pointed out that the reverse current method is more sensitive to temperature variations than the FVM, which can enhance the signal to noise ratio measurements. However, the research community favors forward current instead of reverse current for calibration and information on the practicality of the reverse current method is scarce.

## 5. Other Approaches

### 5.1. Thermocouple Thermometry

Thermocouple thermometry is a widely used contact temperature measurement technique that relies on an intrinsic thermoelectric phenomenon known as the Seebeck effect. In a basic explanation, an electromotive force is generated when the two ends of the thermocouple experience a temperature difference, and the resultant voltage difference between the measurement and the reference junction point can be evaluated to probe the temperature of an unknown point [218]. The usage of passive thermal temperature sensors for thermal characterization may be the simplest and cheapest among other measurement approaches; however, thermocouples cannot be utilized for direct *T_j_* measurement of LEDs since they cannot reach the active area. Thus, researchers should select another reachable point that is close to the chip (e.g., surface, solder point, or electrodes) and assess the temperature based on the thermal resistance from that point to the junction region [219,220] (refer to Figure 1).

Jung and Lee [221] implemented the solder temperature measurement technique to analyze the heat dissipation performance of an LED headlight and evaluated the junction to solder point resistance (R_j-sp_) as 1.7 °C/W. In their analysis, T-type thermocouples were attached to the solder point of the LED chip, and the soldering point temperature (T_s_) at thermal equilibrium was recorded as 62.8 °C. From the solder point temperature measurement analysis, *T_j_* was calculated to be 103.6 °C, which was found to be 6.4 °C less than the FE analysis. Song et al. [222] determined the *T_j_* of a CREE XR-E LED mounted on a thermoelectric cooler using the solder point temperature measurement technique. A T-type thermocouple was directly mounted on the solder point surface, and the R_j-sp_ of the LED was assumed to be 8 °C/W which was taken from the manufacturers’ datasheets. As a result, T_s_ was found to change between 65 and 125 °C with a constant interval of 15 °C for input currents ranging from 300 to 1000 mA with a constant interval of 100 mA. In another study, Faranda et al. [223] tested the heat dissipation performance of refrigerating fluid on a fabricated LED prototype by analyzing the decrease in *T_j_* of a COB white LED. Thermal resistance from the selected measurement point to the junction was given as 6.5 °C/W, and measurements were carried out with a FLUKE 54II thermometer and a temperature sensor. *T_j_* was found to change between 50–56.5 °C and 110.7 °C–123.2 °C for different refrigerating liquid levels. In a more recent study conducted by Rammohan et al. [224], the *T_j_* of a high power LED array was determined using a solder point temperature measurement technique. K-type thermocouples were attached to the solder points of each LED, and the total R_j-sp_ of the LED array consisting of six high power LEDs was given as 2.75 °C/W at 31 °C ambient temperature. Experimental *T_j_* values were found to range between 45 and 85.5 °C for different input powers and ambient conditions. Correspondingly, the authors concluded that thermal imaging temperature maps were in agreement with the reference numerical work findings and solder point temperature measurements.

Direct attachment of a thermocouple tip onto the new generation micro-/nanoscale electronic devices is a handicap [225], and special care has to be taken while preparing a functional setup that can probe temperature in small features. Nowadays, thermocouple detectors manufactured through lithography and vapor-deposition are gaining popularity for thermal characterization in micron and nanoscale electronic devices [226]. A thermocouple probe with a junction size as small as 100 nm^2^ may be produced with the previously mentioned techniques for use in the electronics industry [227]. However, prior fabrication of electric connections with external circuits on micro-LEDs is required to perform temperature measurements with miniaturized thermocouples. Correspondingly, the precise implementation of microthermocouples into the device is considered a time-consuming approach. It is only ideal for one-time measurement of a few discrete points rather than thermal probing on a regular basis. Nonetheless, LED temperature measurement with micro–nano size thermocouples and microsensors has been exercised by several researchers.

Shih et al. [228] micromachined monolithic thermocouples with 78 and 118 μm probe sizes for electrical and thermal inspection of micro-LEDs. Mechanical tests verified that the probe tips could exactly contact the micro-LED electrodes with low contact forces, and, consequently, thermal and electrical properties were successfully determined. Microthermocouples were also adopted in the study of Xiao et al. [142], and it seems that due to the direct covering of the surface of the LED, light absorption both at the surface of the thermocouple and LED or light reflection back to the active region resulted in the overestimation in temperature findings. Although the authors claimed that the 200 μm diameter thermocouple surface was barely affected by the incident or reflected light, the effect of light emission blockage on the LED itself was not discussed. In a more recent study, Choi et al. [229] fabricated a Pt-based microscale resistance temperature sensor with a lift-off process and embedded the sensor onto an SMD-LED package for *T_j_* measurement. Compared to the unstable microthermocouples, the stationary position of the developed Pt sensor was claimed to minimize error by providing simple and reliable thermal characterization. Even if a good agreement between the microsensor measured *T_j_* temperature of the SMD-LED and the numerical and structural thermal analysis is observed, prior calibration of the temperature coefficient of the resistance of the Pt microsensor via another reliable measurement approach is still required for accurate thermal characterization.

In summary, the usage of thermocouples for temperature monitoring is relatively straightforward. They provide rapid response, easy maintenance, and cost-effective solutions for many applications. However, *T_j_* measurements with thermocouples are highly dependent on the thermal resistance from the measurement point to the junction point. Generally, those values are acquired from manufacturer datasheets that represent average resistance values only, and the additional contact resistance between the tip of the thermocouple and the point of interest is generally ignored. The solder point temperature technique is only applicable for simple LED configurations in which the LED is solely mounted over a PCB. In most cases, reaching the solder point of complex LED packages and lamps is a challenging process, and soldering or the usage of adhesives to attach thermocouples to joint points can damage the package integrity and decrease the accuracy of the technique. The spatial resolution and response time of the thermocouple thermometry method are limited by the probe size and thermal capacitance of the thermocouple, respectively. Moreover, light absorption-induced thermocouple self-heating due to local luminance cannot be disregarded since it might lead to the overestimation of *T_j_* during operation [230].

### 5.2. Magnetic Nanoparticle Thermometry (MNPT)

In recent years, the imaging of magnetic nanoparticles at molecular concentrations for temperature measurements has been emerging in micro- and nanoscale thermal investigations [231,232,233,234,235]. Magnetic nanoparticles are super-paramagnetic substances that have a temperature sensitive magnetization curve that allows them to be used for nanosecond-resolved internal temperature probing. Even though magnetic nanoparticle thermometry (MNPT) usage is currently more common in biomedical and biological applications, Hu et al. [236] employed the MNPT technique to determine the heating and cooling characteristics of LEDs. In their analysis, the blue LEDs’ chip surfaces were coated with a layer of MNP, and an AC magnetic field generator and magnetic field detector were used to excite and collect signals from the sample. Particle influence over light extraction was tested with an integrated sphere system to ensure that the MNPs did not alter the optical performance. A one-to-one mapping relation between the magnetization intensity and temperature was formed according to the Langevin equation. Before the LEDs’ thermal analysis, the system was calibrated with thermocouples and ferrofluid samples whose temperature was known. According to the first and third magnetic field harmonic responses, the LED temperatures were recorded to be 31.2, 41.3, and 53.8 °C when operating at 25 °C ambient temperature and under 5.0, 5.1, and 5.2 V input voltages. Although the proposed MNPT technique is a promising new approach in the field of experimental *T_j_* measurement of LEDs, the technique failed to provide detailed point to point temperature profiles of the surface of the LED. Additionally, similar to the other measurement techniques that utilize responsive particles and coatings (Raman active particles and liquid crystals), the measured temperature belongs to the MNP layer temperature, which is assumed to be equal to the LED chip surface temperature and not the actual LED *T_j_*.

## 6. Summary and Conclusions

The influence of the thermal issues is preventing LEDs from reaching their true potential. Especially, generated heat in the p-n junction region is a direct indicator of poor performance that results in a decrease in radiant flux, light quality, efficiency, and reliability. Considering the challenge of the development of an adequate cooling architecture, interpretation of photometric characteristics, and package capabilities to overcome the thermal issues, it is of fundamental importance to accurately determine *T_j_* in the actual operating environment. Correspondingly, in this critical review, we summarized a large number of experimental LED *T_j_* measurement approaches to address the measurement principle, accuracy, and applicability of the methods for various types of LEDs. In addition to the critical discussions provided in this paper, possible problems one may experience with each measurement approach were also explored to introduce a helpful guideline for experimental research on LEDs. The provided comparisons of the typical experimental parameters of work of different researchers are meant to be a useful reference for future research in this field. A summary of the key advantages and limitations of each measurement method is as Table 5:

## Figures and Tables

**Table 1:** TSOP-based experimental temperature measurement parameters that are employed in the literature for the thermal characterization of LEDs. References are sorted by year. Not reported parameters are indicated by a hyphen in the table. (Δδ: uncertainty, λ: wavelength).

		LED Type		Temperature Coefficient		Measurement Range				
Authors	Year	Color	Material System	λ_Peak_ (nm/K)	λ_FVHM_ (nm/K)	T (K)	λ_Peak_ (nm)	λ_Centroid_ (nm)	λ_FWHM_ (nm)	Δδ (K)
Hong and N. Narendran [65]	2004	Red	AlGaInP	0.1376	-	298–343	627–640	-	-	-
Chhajed et al. [77]	2005	Red	AlGaInP	0.1562	0.1812	295–393	629–644	-	19.2–30.8	10
		Blue	GaInN	0.0389	0.0466		460–464		23.9–26.7	
		Green	GaInN	0.0308	0.0625		517–519		46.6–50.4	
Lin et al. [70]	2012	Blue	-	0.03181	0.0532	298–338	453–454.5	-	19.3–22	-
Chen and Narendran [83]	2013	Amber	AlGaInP	-	0.052	295–338	591–597	-	-	-
Chen et al. [85]	2014	White	-	0.085	-	300–370	450.5–456.5	-	-	-
Arik et al. [63]	2014	White	InGaN	0.04	-	298–363	-	-	-	-
Priante et al. [94]	2018	UV	AlGaN-NanoWire	0.027	-	298–358	-	-	-	-
Jin et al. [89]	2019	Red	AlGaInP	0.108	-	298–348	-	650.3–655.5	-	0.9
		Blue	GaInN	0.026			-	455.4–456.7		
		Green	GaInN	0.025			-	531.7–523.9		

**Table 2:** IR thermography parametric summary for the employed parameters in the literature for the temperature measurement of LEDs. References are sorted by year. Not reported parameters are indicated by a hyphen in the table. (ε: emissivity).

		LED Type			Resolution		
Authors	Year	Color	Material System	ε	Spatial (µm or mrad)	Thermal (K)	Spectral Range (μm)
Mashkov et al. [108]	2010	White	-	-	-	0.1	8–13
Corfa et al. [104]	2010	White	-	0.27 (White LED)	-	-	2.5–5.0
Wu et al. [66]	2012	Blue	-	0.5 (GaN) 0.89 (Sapphire) 0.02 (Au Electrode) 0.05 (Al Heat Slug)	1.07 mrad	0.05	2–15
Cheng et al. [114]	2012	Green	InGaN	0.90–0.94 (LEDs) 0.92 (Molding)	468	0.02	-
		Blue	InGaN				
		Red	AlGaInP				
Chang et al. [107]	2012	Green	InGaN/GaN MQW	0.82 (GaN) 0.51 (ITO) 0.25 (Contact Metal) 0.96 (Black Paint)	15	0.03	3.5–5.1
Chernyakov et al. [116]	2013	Blue	InGaN/GaN	-	3	2	2.5–3
Arik et al. [63]	2014	White	InGaN	0.86 (Sapphire)	30	-	8–9
Priante et al. [94]	2018	UV	AlGaN-NanoWire	-	-	-	7–14
Jin et al. [89]	2019	Red	AlGaInP	-	30	-	-
		Green	GaInN		31		
		Blue	GaInN		32		
Ozluk et al. [115]	2019	Green	InGaN	0.84 (GaN)	-	1	2.5–5.1
Aladov et al. [117]	2019	Blue	AlInGaN	-	3	0.2	5–12
Muslu et al. [129]	2021	Red	AlGaInP	0.84 (GaN)	-	-	-

**Table 3:** Raman spectroscopy parameters that are employed in the literature for *T_j_* measurement of LEDs. References are sorted by year. Not reported parameters are indicated by a hyphen in the table. (λ: wavelength).

		LED Type				
Author	Year	Color	Material System	λ_Peak_ (nm)	λ_Source_ (nm)	Resolution (μm)
Schwegler et al. [156]	1999	UV	InGaN/GaN	410	514	0.7
Chitnis et al. [155]	2002	UV	AlGaN/AlGaN	324	488	-
Senawiratne et al. [160]	2008	Blue	GaInN/GaN	-	785	-
		Green				
Wang et al. [162]	2010	UV	InGaN/AlGaN	370	442	5
Natarajan et al. [169]	2013	UV	AlGaN/GaN MQW	-	488	1
Horiuchi et al. [168]	2015	Blue	-	-	632.8	-
Tamdogan et al. [164]	2018	Blue	-	465	532, 440, and 325	1
		White		-		
Park et al. [163]	2018	Blue	InGaN/GaN MQW	448	532	1

**Table 4:** FVM parameters that are employed in the literature for *T_j_* measurement of LEDs. References are sorted by year. Not reported parameters are indicated by a hyphen in the table. (*I_p_*: pulse current, t_p,max_: maximum pulse duration time, *V_f_*: forward voltage, and Δδ: uncertainty).

Authors	Year	Color	Material System	Temperature Coefficient of *V_f_* (mV/K)	Behavior of *V_f_*–*T_j_*	I_p_ Range (mA)	t_p,max_ (μs)	Operating *V_f_* (V)	Δδ (K)
Xi et al. [62]	2005	UV	AlGaN	−5.8	Linear	10–50	-	5.2–7.7	3
Xi et al. [64]	2005	UV	AlGaN	−5.8	Linear	10–100	-	-	3
			GaInN	−2.3					
Jiang et al. [186]	2006	Blue	InGaN/GaN	−3	Linear	20–100	-	-	-
		Blue	InGaN/GaN	−2.7					
Keppens et al. [184]	2008	Red	AlGaInP	−3.391	Linear and Non-linear	0.01–100	10,000	3.408	-
		Red	AlGaInP	−2.998				2.58	
		White	-	−2.219				3.246	
		Green	InGaN MQW	−1.767				2.77	
		White	-	−1.742				2.98	
		Blue	InGaN MQW	−1.653				2.981	
		White	-	−1.648				3.017	
		Green	InGaN MQW	−1.466				2.541	
		White	-	−1.423				2.982	
		White	-	−1.263				2.887	
Cheng et al. [114]	2012	Green	-	−2.714	Linear	0.1	-	2.39–2.58	4.8
		Blue		−2				2.32–2.46	4.3
		Red		−1.714				1.54–1.66	3.9
Meyaard et al. [187]	2013	Blue	GaInN	−1.7	Linear and Non-linear	-	-	-	-
Liu et al. [203]	2014	Blue	-	−1.043	Linear	10	-	-	0.01
Oh et al. [196]	2017	Blue	GaInN/GaN MQW	−1.74	Linear	100	500	2.86–3.2	-
Priante et al. [94]	2018	UV	AlGaN-NanoWire	−6.3	Linear	1	25	-	-
		UV		−5.2					
Lee et al. [111]	2018	White	-	−2.17	Linear	0.1	-	-	-
Tamdogan et al. [164]	2018	Blue	-	−1.6	Linear	1	1000	2.45–3.5	-
		White							
Ozluk et al. [115]	2019	Green	InGaN	−3.12	Linear	1	1000	2.328–2.395	2.3
Zheng et al. [145]	2021	Blue	InGan/GaN	−2.37	Linear and Non-linear	0.1	-	-	-
Onwukaeme et al. [188]	2021	Blue	InGaN/GaN	−1.97 to −4.34	Non-linear	20	100	-	3
Choi et al. [211]	2021	White	-	−1.58	Linear	0.1	-	-	-

**Table 5:** Summary of the LED *T_j_* measurement methods.

Measurement Method	Measurement Principle	Measurement Location	Advantages	Limitations
TSOP	Spectral Power Distribution	*T_j_*	▪ Good spatial resolution ▪ No contact ▪ Direct indicator of *T_j_*	▪ Requires expensive measurement devices ▪ Requires an unobstructed view of the surface ▪ Complex relation between temperature and the optical parameters
IR Thermography	Radiation	Unclear (Surface, bottom, etc.)	▪ Rapid response ▪ Provides temperature maps ▪ No contact	▪ Limited spatial resolution ▪ Measurement accuracy highly dependent on the ambient conditions and emissivity ▪ Calibration is necessary ▪ May require an unobstructed view of the device
Thermoreflectance	Reflectivity	Interconnections	▪ Good spatial resolution ▪ No contact ▪ Rapid response	▪ Not direct indicator of the *T_j_* ▪ May require an unobstructed view of the device
Raman Spectroscopy	Phonon Frequency	Unclear (Semiconductor, surface, etc.)	▪ Good spatial resolution ▪ No contact (if Raman active particle coatings are not used)	▪ Slow acquisition time ▪ May require an unobstructed view of the device
Liquid Crystal	Hue	Surface	▪ Relatively good spatial resolution ▪ Low cost	▪ Thermal spreading effect disturbs the measurement ▪ Subjective evaluation is necessary ▪ Temperature resolution is limited to the transition temperature of the liquid crystal ▪ Not a direct indicator of the *T_j_*
TSEP	Electrical	*T_j_*	▪ Can be used on packaged LED ▪ No contact ▪ Direct indicator of *T_j_*	▪ Measures the average *T_j_* ▪ Prior calibration of the *V_f_* is needed
Thermocouple Thermometry	Seebeck Effect	Surface or Solder Point	▪ Low cost ▪ Readily available ▪ Fast response	▪ Limited resolution ▪ Requires direct contact and therefore an invasive approach ▪ Not a direct indicator of the *T_j_*
MNP Thermometry	Magnetic Field	Surface	▪ Promising new approach for temperature measurement of non-transparent objects	▪ Limited temperature accuracy ▪ Not a direct indicator of the *T_j_*
